# Enhancing Occupational English Speaking Skills among STEM Students through an Integrated EOP–Ethnopedagogical Digital Framework

**DOI:** 10.12688/f1000research.182353.1

**Published:** 2026-07-11

**Authors:** Liya Astarilla Dede Warman, Hadriana Hadriana, Indah Tri Purwanti, Afrianto Daud

**Affiliations:** 1 Faculty of Engineering and Computer Science, Universitas Sains dan Teknologi Indonesia, Pekanbaru, Riau, 28299, Indonesia; 2 Faculty of Teacher Training and Education, Universitas Riau, Pekanbaru, Riau, 28293, Indonesia

**Keywords:** e-module; English for Occupational Purposes; ethnopedagogy; Riau Malay culture; speaking ability

## Abstract

**Background:**

English for Occupational Purposes (EOP) instruction is critical for equipping university graduates with professional communication competencies required in globalized labor markets. However, EOP materials in non-English major programs often lack connection to learners’ disciplinary contexts and sociocultural backgrounds, limiting pedagogical relevance and efficacy. This study introduces the EOP–Ethnopedagogical Digital (EED) Framework and its implementation through EOP EMPOWER, a web-based e-module grounded in Riau Malay ethnopedagogy.

**Methods:**

A quasi-experimental pretest–posttest control-group design was employed, involving 162 Computer Science students (n = 81 experimental; n = 81 control) from three universities in Riau Province, Indonesia. The experimental group received eight weeks of instruction through EOP EMPOWER, while the control group received conventional instruction. Speaking performance was assessed across five dimensions—pronunciation, grammar, vocabulary, fluency, and comprehension—using a validated rubric (ICC = .92; Cronbach’s α = .832; Aiken’s V = .89). Data were analyzed using paired-samples t-tests, independent-samples t-tests, and one-way ANCOVA for effect size estimation.

**Results:**

The experimental group demonstrated statistically significant improvements following the intervention (p < .001), with a large effect size (η ^2^p = .376), substantially outperforming the control group (post-test M = 71.21 vs. M = 61.57; mean gain = 18.17 vs. 8.54 points). Vocabulary and comprehension showed the most pronounced gains, followed by fluency, grammar, and pronunciation.

**Conclusions:**

EOP EMPOWER significantly enhances occupational English speaking ability among STEM students. The EED Framework demonstrates that culturally embedded digital instruction, grounded in Riau Malay ethnopedagogy and Vygotsky’s sociocultural theory, can effectively mediate occupational language acquisition. The study contributes to the SDG 4 Quality Education agenda by offering an empirically validated, culturally responsive, and replicable pedagogical model applicable across diverse higher education contexts.

## Introduction

Achieving Sustainable Development Goal 4 (SDG 4) inclusive and equitable quality education that promotes lifelong learning opportunities for all requires higher education institutions to develop graduates who are not only technically proficient in their disciplines but also capable of communicating effectively within globalized professional environments ^1^ In line with SDG 4 Target 4.4, which calls for a substantial increase in the number of youth and adults with relevant technical and vocational skills, English proficiency has emerged as an indispensable occupational competency for university graduates, particularly in science and technology disciplines. ^2^ Large enterprises require graduates who are not only competent in their disciplinary fields but also capable of communicating effectively in English within multicultural and multinational professional environments. ^3^ ^,^ ^4^ In high-stakes occupational contexts such as recruitment and selection, the quality of candidates’ communicative experiences, particularly their spoken English, constitutes a critical dimension of professional evaluation. ^5^

Despite growing recognition of occupational speaking competence as an essential graduate attribute, many non-English-major students continue to face significant barriers to English-language development. For Computer Science students in particular, speaking English in real time demands simultaneous management of linguistic, cognitive, and social pressures that exceed those encountered in receptive skills development. ^6^ ^,^ ^7^ Common challenges include difficulties with fluency, grammatical accuracy, limited vocabulary range, and pronunciation errors. ^8^ Beyond linguistic barriers, affective obstacles including speaking anxiety, fear of negative evaluation, and low self-efficacy further impede willingness to communicate. ^9^ ^,^ ^10^

To address these interconnected gaps, this study examines the impact of EOP EMPOWER, an integrated EOP–Ethnopedagogical Digital (EED) Framework instantiated as a web-based e-module grounded in Riau Malay ethnopedagogy, on the speaking ability of Indonesian Computer Science students. The EED Framework positions ethnopedagogical integration not as supplementary cultural decoration but as a constitutive pedagogical mechanism that structures authentic task design, professional scenario construction, and learner identity engagement. The study is guided by the following research questions:

**RQ1:** To what extent does EOP EMPOWER enhance university students’ occupational English speaking ability?

**RQ2:** Which speaking performance dimensions are most significantly influenced by the implementation of EOP EMPOWER?

**Figure 1.**
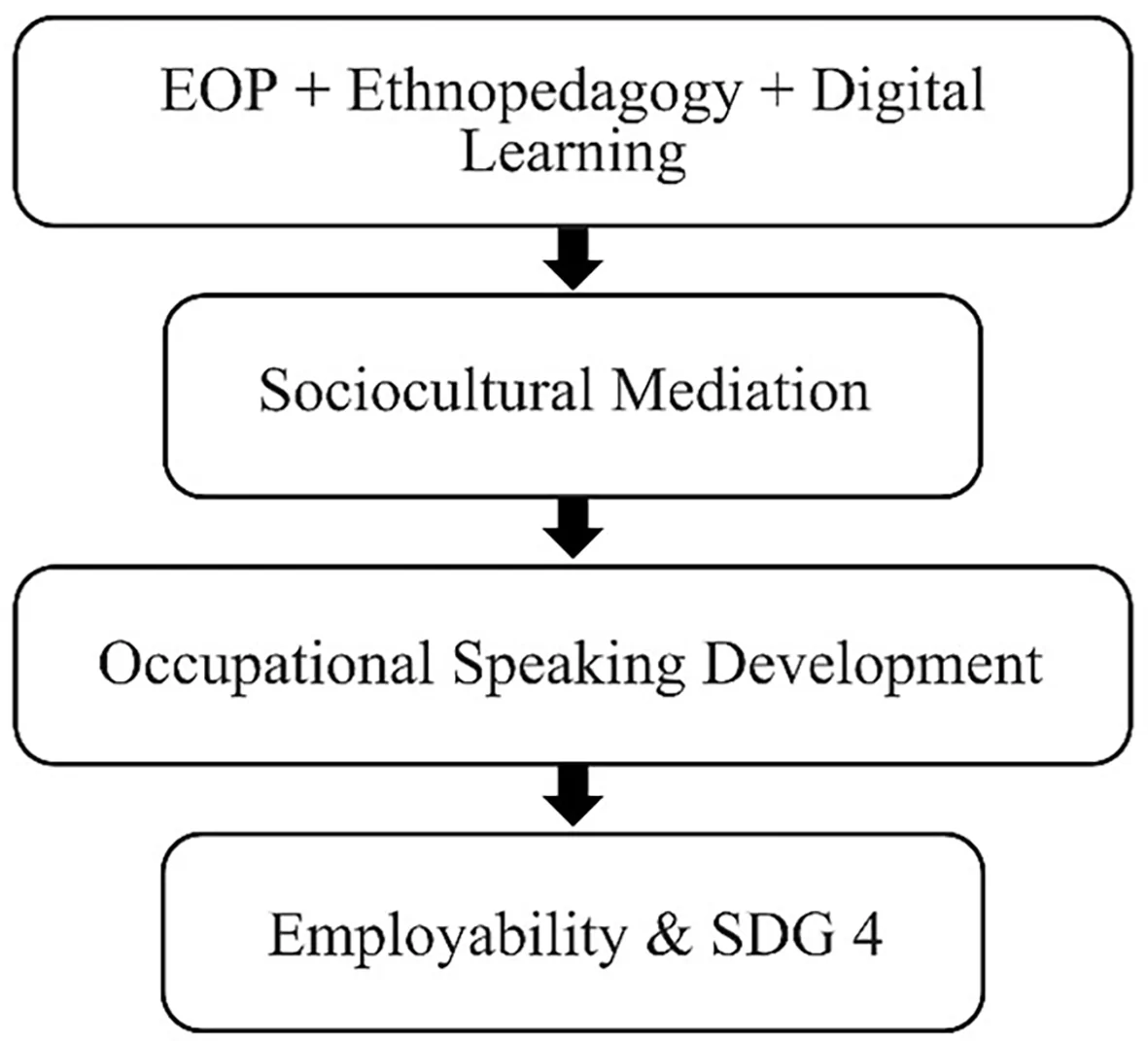
Conceptual Architecture of the EOP–Ethnopedagogical Digital (EED) Framework for Occupational English Speaking Development.

Figure 1 illustrates the conceptual architecture of the EED Framework, which integrates EOP instruction, ethnopedagogical values, and digital learning features through sociocultural mediation mechanisms to enhance occupational English speaking competence.

The framework conceptualizes ethnopedagogy not merely as cultural content, but as an active pedagogical mechanism that shapes learner engagement, contextual communication, and professional identity formation. This study makes three interrelated contributions to the field of occupational language education. First, it introduces the EOP–Ethnopedagogical Digital (EED) Framework as an integrated pedagogical model specifically designed for non-English-major STEM students. Second, it provides large-scale quasi-experimental evidence (n = 162) with rigorous multidimensional speaking assessment across five proficiency dimensions. Third, it extends the theoretical linkage between culturally responsive pedagogy and occupational language acquisition by demonstrating empirically how indigenous cultural values can function as active pedagogical mechanisms in professional English instruction. This study also addresses a critical gap in preparing STEM graduates for global communication demands, contributing directly to the SDG 4 Quality Education agenda.

### English for occupational purposes in higher education

English for Occupational Purposes (EOP) is explicitly designed to prepare learners for professional communication in specific occupational contexts, with particular emphasis on authentic workplace language use. ^11^ ^,^ ^12^ EOP instruction is distinguished by its systematic integration of needs analysis, domain-specific authentic materials, and task-based activities that reflect communicative demands learners will encounter in their future professions. ^13^ ^,^ ^14^ The emergence of digital EOP platforms offering unprecedented flexibility, interactivity, and access to authentic multimedia content represents a transformative opportunity for higher education institutions seeking to deliver pedagogically relevant occupational language instruction aligned with 21st-century workplace demands. ^15^ ^,^ ^16^ Mobile-assisted language learning approaches have further demonstrated efficacy in developing occupational speaking competencies, with EFL learners reporting measurable improvements in fluency and communicative confidence through targeted digital speaking activities. ^17^ ^,^ ^18^

Furthermore, cross-cultural concepts in EOP programs address both linguistic and cultural demands in professional settings. ^13^ EOP is one of the essential competencies that prospective graduates need to master to enhance their employability and readiness to enter the workforce. ^19^ EOP instruction is designed to strengthen students’ practical skills, as they are expected to use English appropriately in specific situations and in their professional fields in the workplace. ^20^ In short, EOP instruction plays a crucial role in preparing students for their future careers by integrating authentic, workplace-oriented, and cross-cultural learning materials that enhance professional language competence and readiness for employment.

### Speaking Ability in Occupational Contexts

Speaking proficiency is one of the most in-demand competencies in modern professional workplaces. In occupational settings, the capacity to communicate orally with clarity, precision, and cultural appropriateness is a fundamental prerequisite for career success. ^21^ ^–^ ^23^ Drawing on established assessment frameworks, ^24^ the key components of speaking proficiency include: (1) fluency natural pace and automaticity; (2) pronunciation intelligible sound production; (3) grammar accurate syntactic structures; (4) vocabulary breadth and contextual appropriateness; and (5) comprehension real-time decoding and response. The simultaneous development of all five dimensions within a single instructional module represents a substantial pedagogical challenge that digital, task-based EOP instruction is uniquely positioned to address through authentic, scaffolded, and iterative speaking practice. ^25^ ^,^ ^26^ Emerging immersive technologies such as virtual reality have further demonstrated effectiveness in developing EFL speaking skills through situated learning approaches that authentically simulate occupational communication contexts. ^27^

Effective workplace speaking requires more than just putting words together clearly; it also means using body language, gestures, and facial expressions to communicate. ^28^ ^,^ ^23^ Students must construct meaning by producing, receiving, and processing information during interaction. In addition, students need to master several aspects of speaking skills grammar, pronunciation, fluency, vocabulary, and comprehension in order to ensure effective and efficient communication. ^24^ ^,^ ^29^ ^,^ ^30^ In other words, effective speaking involves not only clear verbal expression but also the appropriate use of nonverbal communication, and mastery of linguistic components to achieve meaningful and successful interaction.

### Ethnopedagogy of riau malay culture in language learning

Ethnopedagogy is a culturally grounded educational framework that systematically incorporates indigenous knowledge systems and community-based values into formal instructional practice. ^31^ ^–^ ^34^ This orientation finds robust grounding in ^35^ sociocultural theory, which posits that learning is inseparably mediated by cultural tools and interpersonal interaction within specific sociocultural contexts. Ethnopedagogy resonates deeply with the framework of Culturally Responsive Teaching (CRT), which argues that instruction is most effective when it deliberately connects content to students’ sociocultural backgrounds. ^36^ ^,^ ^37^ Riau Malay culture embodies politeness, cooperation, hard work, and self-confidence as foundational professional character values. ^38^ ^,^ ^39^ EOP EMPOWER operationalizes four of these values as active pedagogical tools that shape speaking task design and the construction of authentic professional scenarios. ^40^ Demonstrate that learners who perceive a coherent relationship between the target language and their own cultural identity develop stronger motivation, deeper engagement, and more sustained language learning trajectories. ^41^ ^,^ ^37^ ^,^ ^42^ Riau Malay culture embodies a rich system of identity values including politeness, respect, cooperation, social harmony, and dignity that collectively constitute the ethical and philosophical foundation of the Malay professional character. ^38^ ^,^ ^43^

## Methods

### Research design

**Figure 2.**
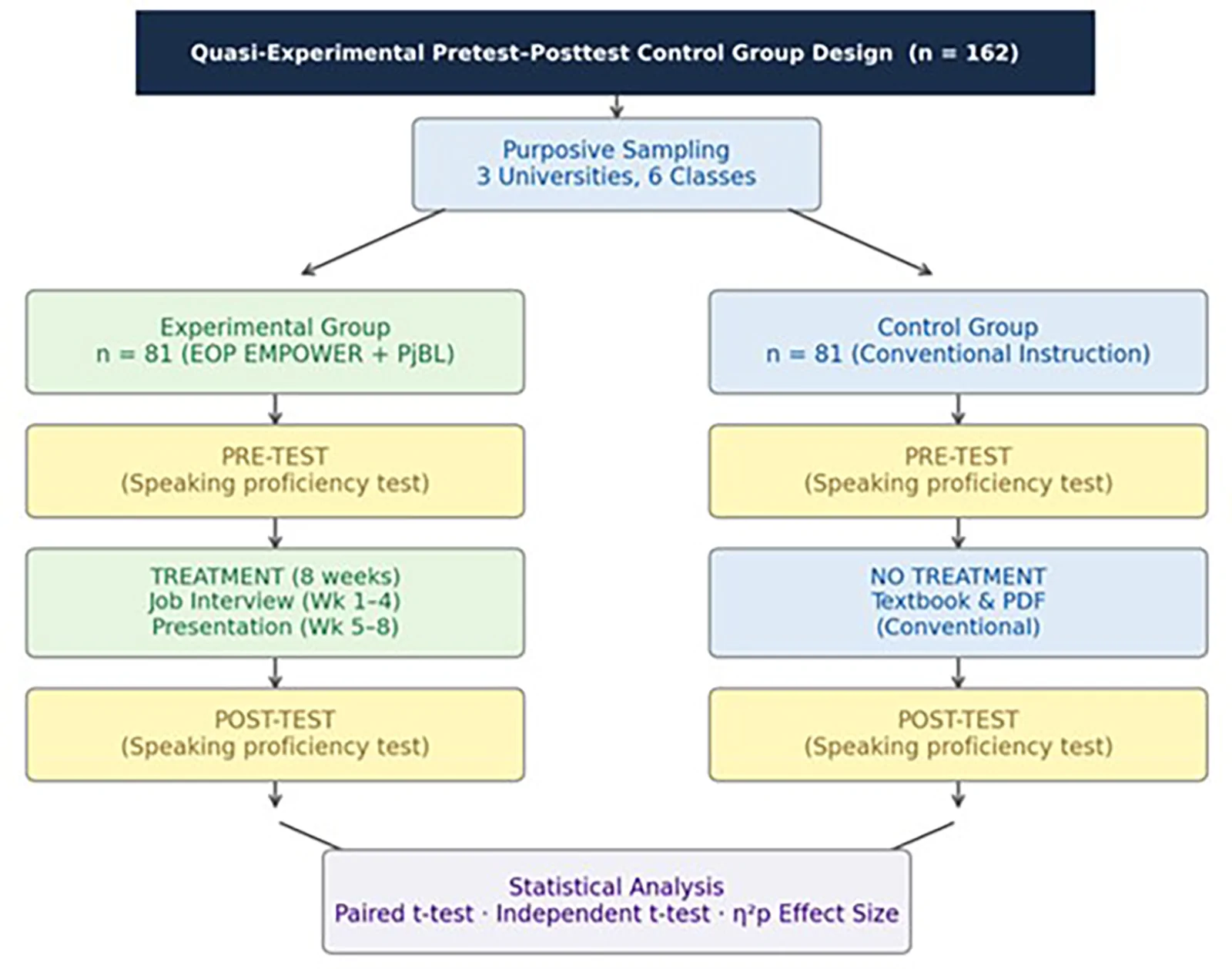
Research design and procedural framework.

This study was conducted over an eight-week instructional period involving both experimental and control groups. Prior to the intervention, all participants completed a speaking pre-test to determine baseline equivalence. The experimental group received instruction through the EOP EMPOWER platform, which integrated occupational English materials, multimedia-based speaking activities, and ethnopedagogical values derived from Riau Malay culture. Students in the experimental group engaged in structured learning activities including video-based presentation tasks, simulated job interviews, collaborative discussions, pronunciation exercises, and culturally contextualized workplace communication practices. The implementation followed the procedural stages illustrated in Figure 2, beginning with orientation and pre-testing, followed by systematic instructional treatment, guided speaking practice, peer interaction, and post-test evaluation. In contrast, the control group received conventional classroom instruction using printed textbooks and PDF-based materials without access to the EOP EMPOWER platform or ethnopedagogical integration. Both groups studied equivalent occupational English topics and were taught over the same instructional duration to maintain procedural consistency. However, only the experimental group experienced digitally mediated interactive learning and culturally responsive speaking activities embedded within the EED Framework.

### Participants

Participants were selected via purposive sampling. ^44^ Selection criteria required Computer Science students currently enrolled in a compulsory English course at universities with equivalent BAN-PT Level B accreditation. All participants were adults aged 18–19 years at the time of data collection. The total sample comprised 162 students: 81 in the experimental group and 81 in the control group ( Table 1). Prior to intervention, both groups demonstrated statistically equivalent baseline speaking proficiency (pre-test: experimental M = 53.05, control M = 53.02; p = .990), confirming baseline equivalence.

**Table 1.** Demographic characteristics of participants.

Group	Gender	n	%	Total
Experimental	Male	61	75.3	81
	Female	20	24.7	
Control	Male	56	69.1	81
	Female	25	30.9	

*Note*: n = number of students per gender; % = percentage within group.

### Instrumentation and data analysis

Speaking proficiency was assessed using ^24^ rubric across five dimensions (pronunciation, grammar, vocabulary, fluency, comprehension), each scored on a five-point scale. Three trained TEFL experts independently rated video-recorded performances. Inter-rater reliability (ICC = .92) and internal consistency (Cronbach’s α = .832) confirmed excellent instrument reliability. Content validity was established via Aiken’s V = .89. Statistical analyses included Kolmogorov–Smirnov normality tests, Levene’s homogeneity tests, paired-samples t-tests, independent-samples t-tests, and one-way ANCOVA for partial eta squared effect size. ^45^

### Instructional intervention: EOP EMPOWER platform

**Figure 3.**
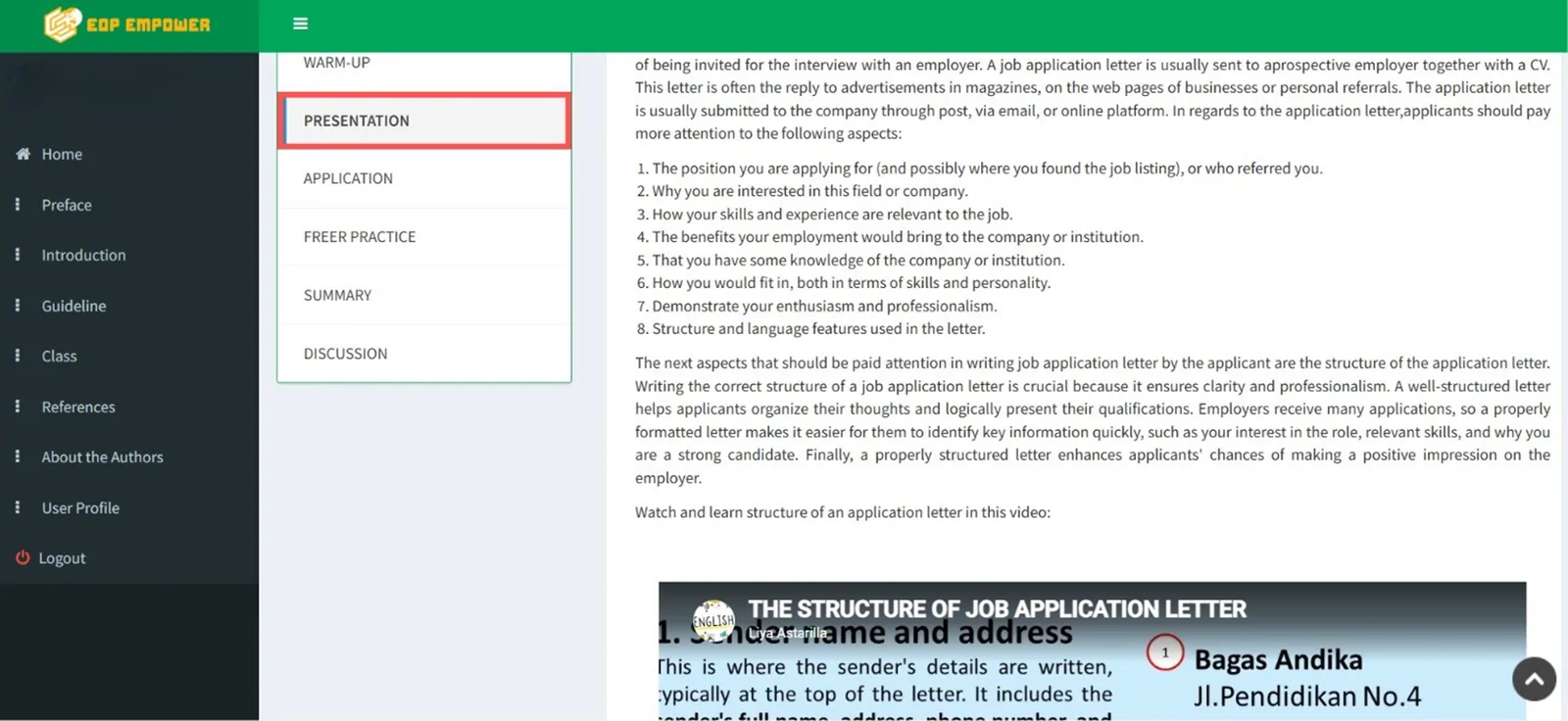
An example of the presentation section of EOP EMPOWER.

**Figure 4.**
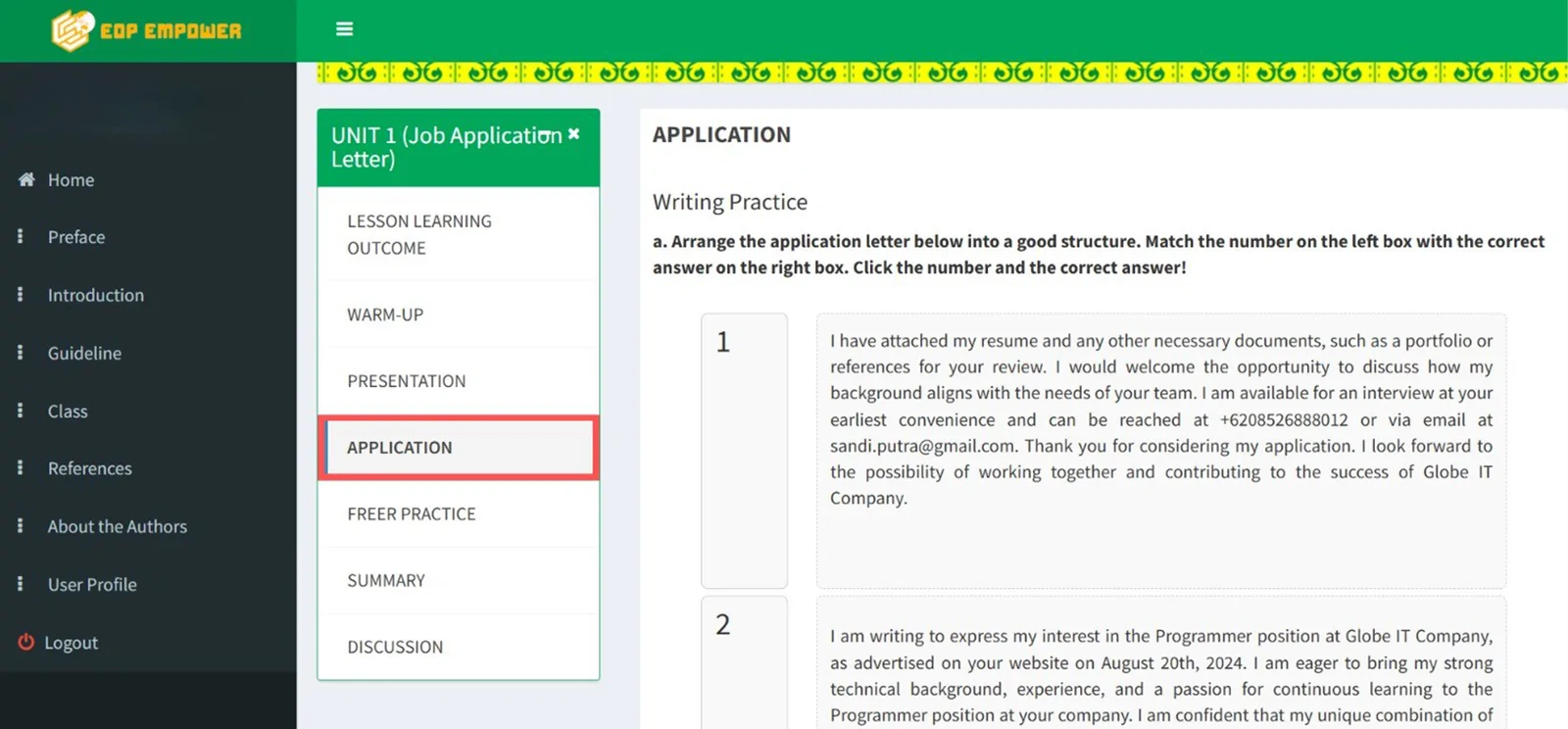
An example of the application section of EOP EMPOWER.

**Figure 5.**
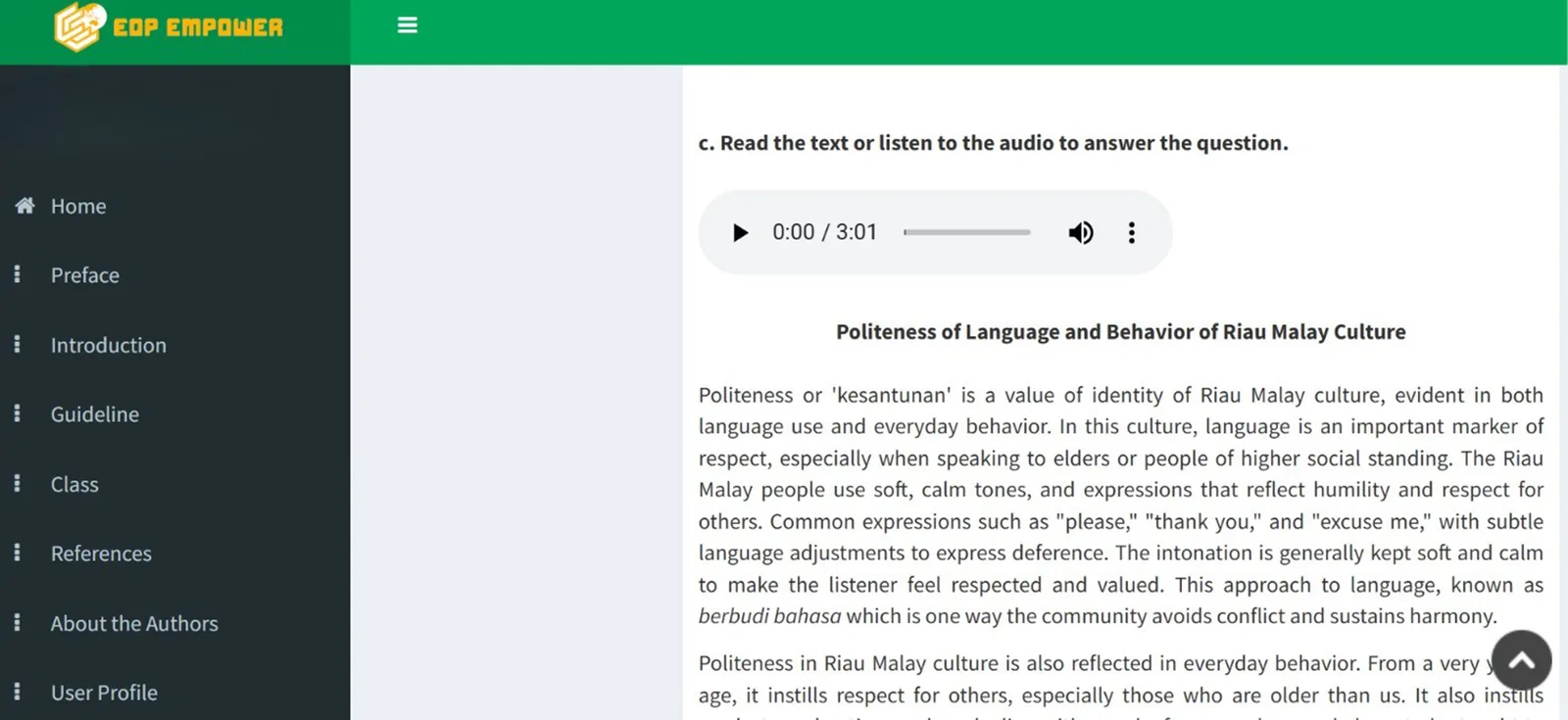
An example of Ethnopedagogy of Riau Malay Culture content in EOP EMPOWER.

EOP EMPOWER is a web-based EOP e-module grounded in Riau Malay ethnopedagogy developed by the authors, accessible via www.eop-empower.com. The platform comprises four structured units, each containing eight sequential learning stages: Lesson Learning Outcome, Warm-Up, Presentation, Application, Freer Practice, Summary, and Discussion. Figure 3 illustrates the Presentation stage, which delivers content through multimedia input, including embedded video. Figure 4 illustrates the Application stage, where learners engage in interactive practices aligned with occupational scenarios. Figure 5 illustrates how students learn and discuss the identity of Riau Malay culture (ethnopedagogy) in the Application stage, specifically politeness, which is relevant to workplace communication.

## Results

This section presents statistical analyses addressing both research questions. Analyses proceed sequentially: assumption checks (normality and homogeneity), within-group improvements (paired t-tests), between-group comparisons (independent t-tests), speaking component analysis, and effect size quantification.

### Assumption checks: Normality and homogeneity

Prior to inferential testing, the normality of the data distribution was examined using the Kolmogorov Smirnov test. Table 2 presents the results of the normality test for both the experimental and control groups across pre-test and post-test scores.

**Table 2.** Results of normality tests (Kolmogorov–Smirnov).

Test	Group	Statistic	df	Sig.
Pre-test	Experimental	.095	81	.067
	Control	.069	81	.200 *
Post-test	Experimental	.075	81	.200 *
	Control	.086	81	.200 *

*Note*: All Sig. values > .05 confirm normal distribution.

^*^ lower bound of true significance.

Following the normality check, the homogeneity of variance between the two groups was examined using Levene’s test. Table 3 presents the results of the homogeneity-of-variance test for both the pre-test and post-test scores.

**Table 3.** Results of homogeneity of variance tests (Levene’s Test).

Test	Levene F	df1	df2	Sig.
Pre-test	.976	1	160	.325
Post-test	1.135	1	160	.288

*Note*: All Sig. values > .05 confirm homogeneity of variance. Parametric tests are justified.

### Descriptive statistics

Table 4 presents the descriptive statistics, including the mean (M) and standard deviation (SD), for both the experimental and control groups across the pre-test and post-test measurements.

**Table 4.** Descriptive statistics for Pre-test and Post-test speaking scores.

Group	Test	M	SD	n
Experimental	Pre-test	53.05	9.27	81
	Post-test	71.21	8.14	
	*Mean Gain*	**18.17**	—	
Control	Pre-test	53.02	9.19	81
	Post-test	61.57	8.36	
	*Mean Gain*	**8.54**	—	

*Note*: M = mean; SD = standard deviation. Shaded rows = Experimental group. Speaking scores range 0–100.

**Figure 6.**
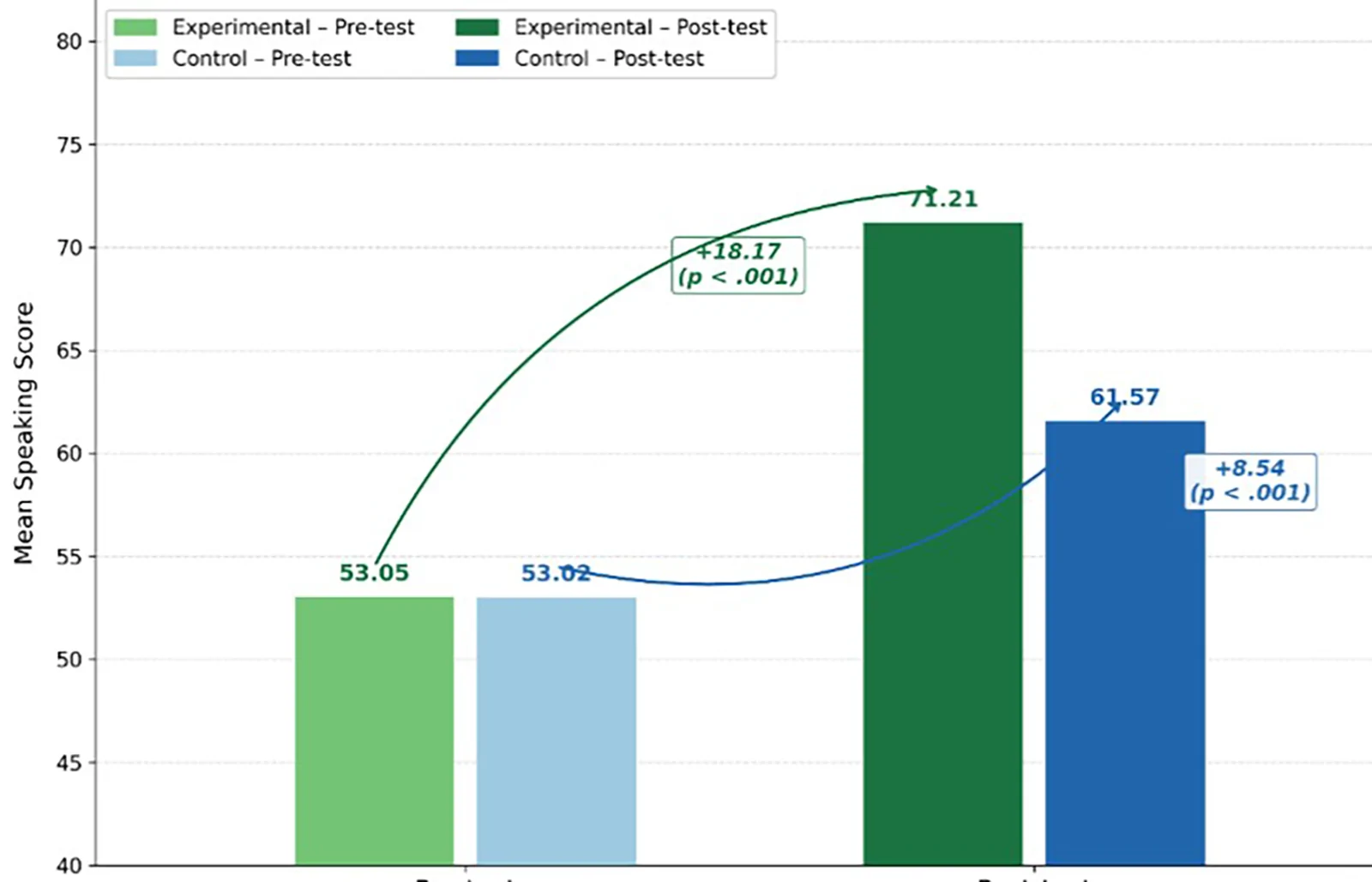
Pre-test and Post-test mean speaking scores by group.

Figure 6 illustrates the pre-test and post-test mean scores for both groups. Both groups had almost identical speaking proficiency before the intervention (Experimental: M = 53.05, SD = 9.27; Control: M = 53.02, SD = 9.19). Following the eight-week intervention, the experimental group demonstrated substantially greater improvement (M = 71.21, SD = 8.14; gain = 18.17 points) than the control group (M = 61.57, SD = 8.36; gain = 8.54 points), with a ratio of 2.13:1.

### RQ1: Within-Group improvement in speaking ability

To address RQ1, a paired-samples t-test was conducted to examine within-group improvement in speaking performance from pre-test to post-test for each group. The results are presented in Table 5.

**Table 5.** Paired-Samples t-Test results.

Group	Mean Diff.	SD	t	Sig. (2-tailed)
Experimental (Pre − Post)	−18.168	8.544	−19.138	.000 ***
Control (Pre − Post)	−8.543	4.847	−15.865	.000 ***

*Note*: df = 80 for both tests. A negative mean difference indicates that the post-test is greater than the pre-test (improvement).

^***^ p < .001.

Table 5 reveals that both groups demonstrated statistically significant within-group improvements from pre-test to post-test (p < .001). The experimental group showed a mean improvement of 18.168 points (t(80) = −19.138, p < .001), while the control group showed a mean improvement of 8.543 points (t(80) = −15.865, p < .001). The magnitude of improvement in the experimental group was more than twice that of the control group, indicating that EOP EMPOWER produced a substantially greater within-group effect on speaking development.

### RQ1: Between-Group comparison of improvement

To further address RQ1, an independent-samples t-test was conducted to determine whether a statistically significant difference existed between the experimental and control groups at both the pre-test and post-test stages. Table 6 presents the results of this between-group comparison.

**Table 6.** Independent-Samples t-Test results.

Test	Levene Sig.	t	df	Sig. (2-tailed)	Mean Diff.
Pre-test	.325	.013	160	.990	.025
Post-test	.288	6.306	160	.000 ***	9.649

*Note*: Equal variances assumed (Levene Sig. > .05). H _0_ rejected for post-test: experimental significantly outperformed control.

^***^ p < .001.

Table 6 shows no significant difference between groups at baseline (p = .990 > .05), confirming equivalent baseline ability. Following the eight-week intervention, a highly significant between-group difference emerged (t(160) = 6.306, p < .001), with the experimental group outperforming the control group by 9.649 mean points, indicating that EOP EMPOWER produced greater improvement in speaking development.

### RQ2: Speaking component analysis

**Figure 7.**
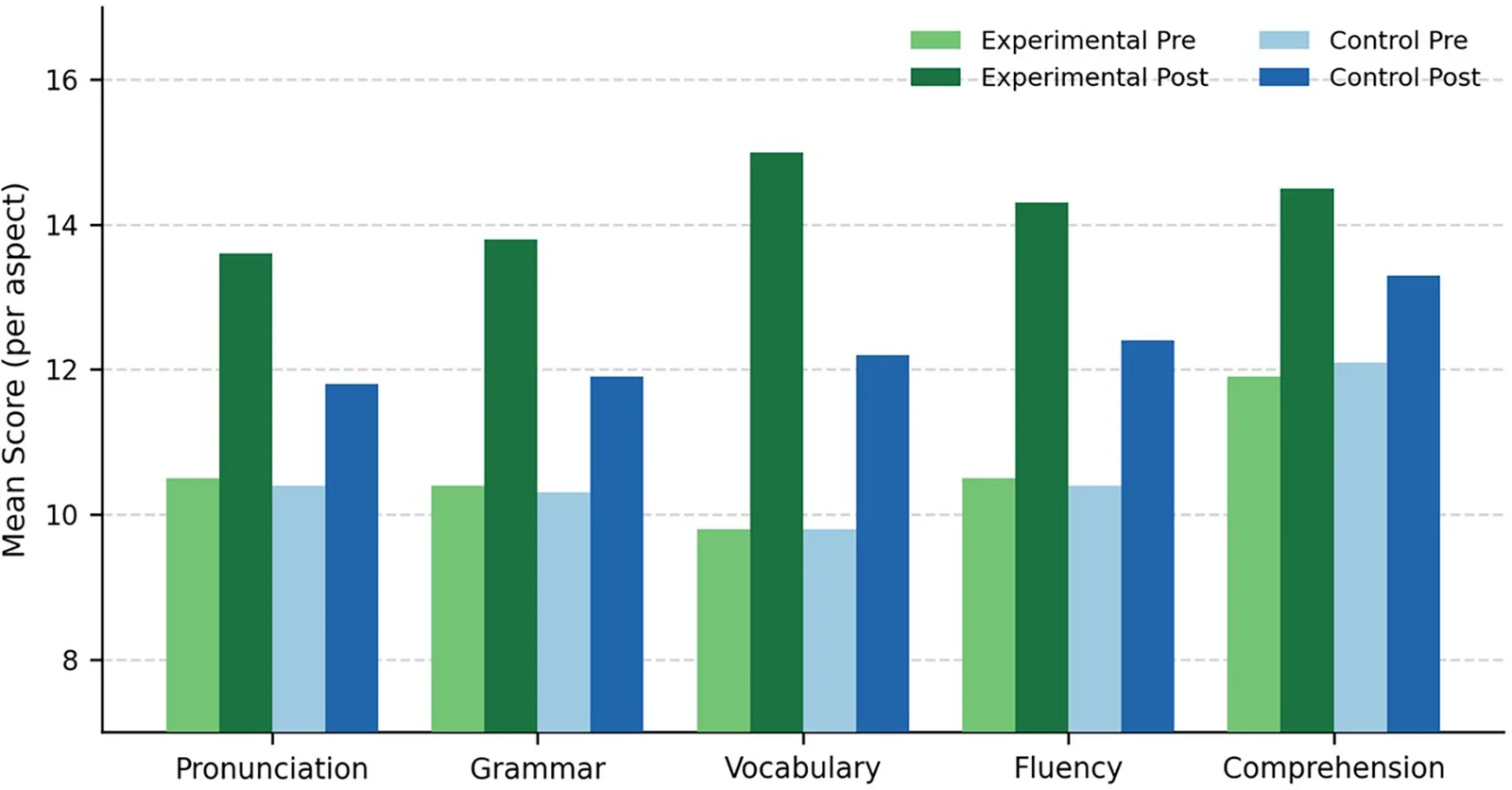
Speaking component scores: Pre-test and Post-test across both groups.

**Figure 8.**
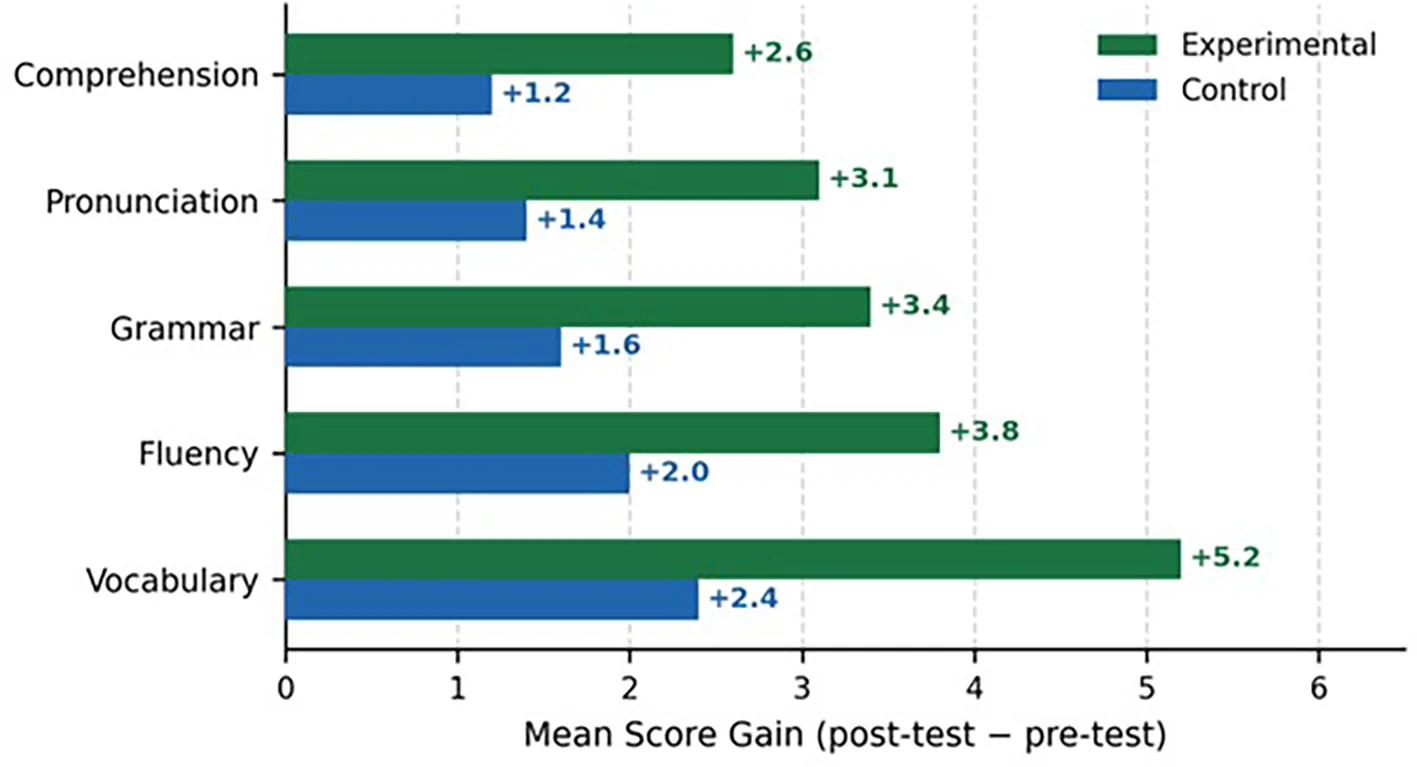
Mean gain score per speaking component.

Figures 7 and 8 present per-component scores and gain scores across both groups. The experimental group achieved greater gains across all five components compared to the control group, with vocabulary (+5.2) and comprehension (+4.4) demonstrating the most pronounced improvements, followed by fluency (+3.8), grammar (+3.4), and pronunciation (+3.1).

The experimental group demonstrated consistently greater improvement across all five components. Figure 7 reveals that the most significant improvements are shown in vocabulary and comprehension, while fluency is also markedly enhanced. These results show that the e-module helps students improve their overall speaking skills, not just sentence structure, but also understanding and communicating ideas.

**Figure 9.**
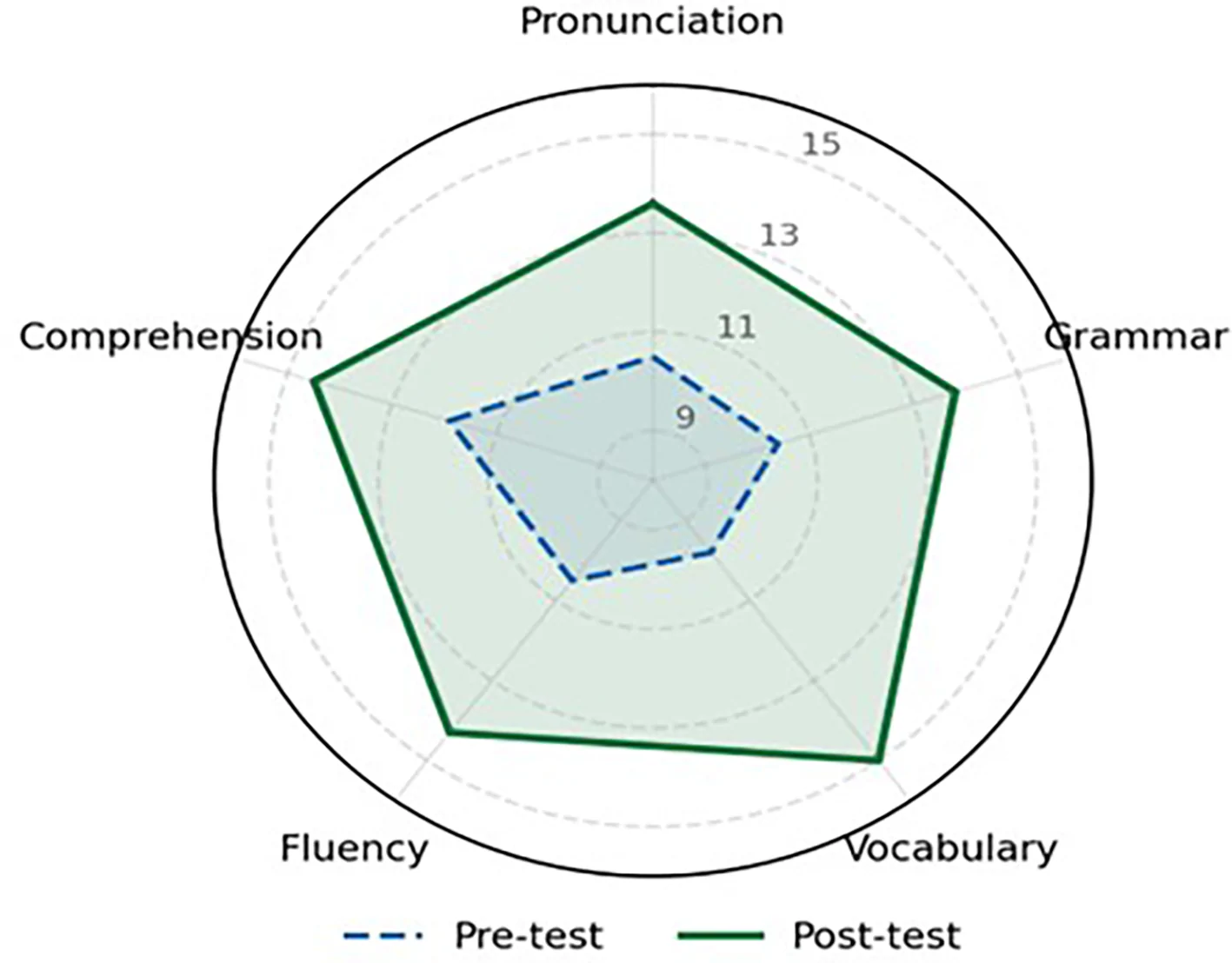
Radar profile of the experimental group: Pre-test vs Post-test.

Figure 9 presents a radar profile comparing the experimental group’s pre-test and post-test performance across all five speaking dimensions. The radar visualization confirms that improvement was balanced and comprehensive across all components, with the post-test profile showing markedly expanded coverage relative to the pre-test baseline, particularly for vocabulary and comprehension. This multidimensional profile reinforces the finding that EOP EMPOWER produced holistic speaking development rather than gains confined to isolated dimensions.

### Effect Size Analysis

To quantify the practical significance of the EOP EMPOWER intervention, a one-way ANCOVA was performed with the pre-test score as the covariate and the post-test score as the dependent variable. Table 7 presents the ANCOVA results, including the partial eta squared (η ^2^p) effect size.

**Table 7.** One-Way ANCOVA Results – Effect Size Analysis.

Source	Type III SS	df	MS	F	Sig.	η ^2^p
Corrected Model	1,672	1	1,672	96.323	.000	.376
Intercept	12,791	1	12,791	736.671	.000	.822
Group	1,672	1	1,672	96.323	.000 ***	.376
Error	2,778	160	.017			
Total	17,241	162				
Corrected Total	4,450	161				

*Note*: R ^2^ = .376 (Adjusted R ^2^ = .372). η ^2^p ≥ .14 classified as large effect (J. Cohen, 1988).

^***^ p < .001.

## Discussion

The findings confirm that EOP EMPOWER significantly and substantively enhances Computer Science students’ occupational English speaking ability, addressing both RQ1 and RQ2 with robust empirical evidence. The experimental group achieved a post-test mean of 71.21, reflecting a gain of 18.17 points from a baseline of 53.05, which substantially exceeded the control group’s gain of 8.54 points. Moreover, the large effect size (η ^2^p = .376) indicates that these differences are not only statistically significant but also pedagogically meaningful. These results extend the evidence base established ^46^ ^,^ ^47^ by providing objective, multi-dimensional assessment data from a larger sample and a more rigorous quasi-experimental design.

Several mechanism-level explanations account for EOP EMPOWER’s superior effectiveness. First, the structured six-stage pedagogical sequence embedded within a PjBL framework ensured progressive scaffolding from controlled to autonomous practice, facilitating the development of automaticity and fluency. ^48^ ^,^ ^49^ Second, the multimedia affordances instructional videos, interactive exercises, and structured peer feedback enhanced motivation and engagement across the eight-week intervention. ^50^ ^,^ ^51^ ^,^ ^52^ Third, the cross-device accessibility of the web-based platform reduced participation barriers for students at regional universities. ^53^ ^,^ ^54^

The finding that vocabulary and comprehension showed the largest gains aligns with the theoretical content characteristics of EOP EMPOWER. The platform’s authentic, discipline-specific speaking scenarios job interview simulations and Computer Science workplace presentations required active deployment of professional technical vocabulary in meaningful communicative contexts, facilitating both lexical consolidation and interactive comprehension development. ^55^ ^,^ ^56^ The integration of Riau Malay ethnopedagogical values as active pedagogical tools likely reduced speaking anxiety and increased willingness to communicate effects consistent with Gay’s (2018) CRT framework and identity investment theory. ^41^ ^,^ ^40^ These findings collectively position EOP EMPOWER as a replicable model of culturally responsive, digitally accessible, and employability-oriented language education that advances the SDG 4 Quality Education agenda.

From a theoretical standpoint, these findings extend ^35^ sociocultural theory by providing empirical evidence that culturally embedded digital instruction can serve as a potent mediating tool that accelerates occupational language acquisition. The EED Framework operationalizes Vygotsky’s concept of cultural mediation at the level of instructional design: indigenous identity values serve as culturally meaningful scaffolds that facilitate learners’ transition from linguistic awareness to professional communicative competence.

Unlike prior studies that have focused either on digital EOP instruction or on culturally responsive pedagogy in isolation, this study integrates both dimensions into a unified, theoretically grounded instructional model. The EED Framework bridges the dual gap between digital EOP research and ethnopedagogy scholarship, offering a design architecture that is simultaneously culturally situated and pedagogically rigorous. Moreover, the EED Framework demonstrates transferability to other multilingual and multicultural higher education contexts beyond Indonesia, where indigenous cultural values and the demands of occupational English coexist as potentially synergistic educational priorities.

### Limitations and Future Research

While this study presents significant findings, several limitations warrant consideration. First, the sample was drawn from three universities within a single Indonesian province, constraining external generalizability. Second, the intervention addressed only two professional scenarios (job interviews and presentations) over eight weeks, not fully capturing the range of communicative demands that Computer Science graduates encounter. Third, the current implementation incorporated four of the twenty-nine Riau Malay identity values; future research should explore the differential contributions of additional values to professional speaking development. Fourth, and importantly, the current design does not isolate the independent effects of the ethnopedagogical integration and the digital learning components within the EED Framework.

Future researchers are recommended to replicate and extend this study across geographically, culturally, and institutionally diverse university settings. Longer intervention durations would facilitate the development of advanced speaking sub-skills requiring sustained practice, such as critical argumentation and extended professional discourse. Mixed-methods designs incorporating qualitative data could further illuminate the mechanisms through which ethnopedagogical integration enhances speaking development and professional identity formation.

## Conclusions

This study provides empirical evidence suggesting that EOP EMPOWER significantly enhances Indonesian Computer Science students’ occupational English speaking ability. Over an eight-week quasi-experimental intervention involving 162 students across three universities, the experimental group demonstrated substantially greater improvement across all five speaking dimensions than the control group (post-test M = 71.21 vs. M = 61.57, p < .001, η ^2^p = .376). Vocabulary and comprehension exhibited the most pronounced gains, reflecting the authentic, content-rich professional communication tasks embedded within EOP EMPOWER. The large effect size (η ^2^p = .376 > .14) confirms strong practical significance. This study contributes to the SDG 4 Quality Education agenda by demonstrating that culturally responsive, digitally accessible EOP instruction can simultaneously advance occupational English proficiency and affirm cultural identity, providing a replicable model applicable across diverse regional and disciplinary contexts in Indonesian and analogous higher education settings.

## Ethical considerations and Informed Consent

This study was conducted in accordance with the ethical principles for research involving human participants. Ethical approval was obtained from the Research Ethics Committee of the Faculty of Teacher Training and Education, Universitas Riau, Indonesia (Approval No: 2282/UN19.5.1.1.5/TD.06/2024; Date: 24 July 2024). Written informed consent was obtained from all participants prior to data collection. Participation was voluntary, and participants were informed of their right to withdraw from the study at any time without penalty. All collected data were anonymized and treated confidentially to protect participants’ privacy.

## Data Availability

Zenodo. Dataset for: Enhancing Occupational English Speaking Skills among STEM Students through an Integrated EOP–Ethnopedagogical Digital Framework. https://doi.org/10.5281/zenodo.20357296. ^57^ This project contains the following underlying data:
•
01_raw_speaking_scores.csv •
02_summary_descriptive_statistics.csv •
03_normality_test_results.csv •
04_homogeneity_test_results.csv •
05_paired_ttest_results.csv •
06_independent_ttest_results.csv •
07_ancova_effect_size_results.csv •
08_speaking_component_analysis.csv •
09_participant_demographics.csv •
EOP_EMPOWER_Dataset_Zenodo.xlsx • Speaking test Instrument.docx • Speaking test Instrument.xlsx Data is available under the terms of the Creative Commons Attribution 4.0 (CC-BY 4.0) licence.
