# Supplementary material for: Enhancing Occupational English Speaking Skills among STEM Students through an Integrated EOP–Ethnopedagogical Digital Framework
Source: F1000Res. 2026 Jul 11;15:1132. [Version 1] doi: 10.12688/f1000research.182353.1 (PMC13560037; doi:10.12688/f1000research.182353.1)
Supplement: Peer review history

# Reviewer report: Reviewer response for version 1

**DOI:** 10.5256/f1000research.201285.r506450

**Published:** 2026-08-11

**Authors:** Prima Aswirna

**Affiliations:** 1 Universitas Islam Negeri Imam Bonjol Padang, Padang, Indonesia

Peer Review Report

**1. Article Summary**

 The manuscript titled *"Enhancing Occupational English Speaking Skills among STEM Students through an Integrated EOP-Ethnopedagogical Digital Framework"* presents a quasi-experimental study evaluated across an 8-week intervention involving 162 Computer Science students ($n=81$ experimental; $n=81$ control) from three Indonesian universities.

 The experimental group used **EOP EMPOWER**, a web-based e-module integrating English for Occupational Purposes (EOP), digital learning tools, and Riau Malay ethnopedagogy (such as politeness and identity values). The control group received conventional textbook/PDF-based instruction. Results showed statistically significant improvements for the experimental group ($p < .001$) with a large effect size ($\eta_p^2 = .376$), outperforming the control group across all five speaking dimensions (pronunciation, grammar, vocabulary, fluency, and comprehension). The study successfully establishes how culturally responsive digital frameworks can enhance occupational communication skills and align with SDG 4 (Quality Education) goals.

**2. Evaluation of Key Criteria**

- **Presentation & Literature Integration:** The work is clearly structured, academically sound, and well-contextualized within contemporary EOP, mobile/digital learning, and culturally responsive teaching literature.
- **Methodology & Soundness:** The quasi-experimental pretest-posttest control-group design is well-executed. The authors confirmed baseline equivalence prior to the intervention ($p = .990$).
- **Replication & Method Details:** Methodological steps, timeframes, sampling criteria, and statistical tests are explicitly detailed. Open dataset details are made publicly accessible via Zenodo.
- **Statistical Analysis:** The statistical tests—including Kolmogorov-Smirnov normality checks, Levene’s tests, paired/independent $t$-tests, and ANCOVA—are appropriate, accurately conducted, and correctly interpreted.
- **Data Reproducibility:** Underlying raw and summary data (CSVs, score sheets, and instruments) are deposited in Zenodo with a CC-BY 4.0 license.
- **Conclusions:** Conclusions directly reflect the empirical findings and are supported by the ANCOVA and per-component gain analyses.

**3. Constructive Feedback & Recommendations for Improvement**

 While the paper is scientifically sound, the following minor revisions are recommended to further improve transparency and clarity prior to final archiving:

1. **Clarify National Contextual Terms:** Terms like "BAN-PT Level B" should be briefly defined for an international audience (e.g., specifying that BAN-PT refers to Indonesia's National Accreditation Agency for Higher Education).
2. **Assessment Delivery Mode Detail:** Clarify whether the rated speaking performances were recorded synchronously (live online interviews/presentations) or asynchronously (pre-recorded video uploads). This detail provides context regarding performance anxiety and cognitive load.
3. **Operationalization of Cultural Values:** Provide a brief summary table or outline detailing how each of the incorporated Riau Malay values (e.g., politeness/'kesantunan', cooperation) directly maps onto specific EOP tasks in the module.
4. **Scope of Framework Variables:** Acknowledge in the discussion/limitations section that while the integrated framework demonstrates high efficacy, the study design does not statistically isolate the individual contribution of the digital medium versus the ethnopedagogical content.

**My study aligns across five key research domains:**

 1. English for Occupational Purposes (EOP) & English for Specific Purposes (ESP)Relevance: The manuscript focuses on preparing non-English majors—specifically Computer Science/STEM students—for professional workplace communication (e.g., job interviews and technical presentations).Connection: If your research involves workplace language needs analysis, ESP/EOP course design, or targeted oral communication skills, this study provides empirical support for integrating discipline-specific task design into vocational and technical language instruction.

 2. Ethnopedagogy & Culturally Responsive Teaching (CRT)Relevance: The EED Framework incorporates local cultural values (Riau Malay ethnopedagogy, such as kesantunan or politeness) directly into instructional tasks.Connection: If your work explores local wisdom, indigenous knowledge systems, or culturally responsive pedagogy, this article demonstrates how cultural identity acts as a mediating tool (grounded in Vygotsky’s sociocultural theory) to reduce speaking anxiety and improve learner engagement.

 3. Digital Pedagogy, Educational Technology, & E-ModulesRelevance: The intervention relies on EOP EMPOWER, a web-based e-module featuring interactive practices, video inputs, and multimedia-assisted learning.Connection: If your focus is on CALL (Computer-Assisted Language Learning), MALL (Mobile-Assisted Language Learning), e-module development, or blended learning design, this study offers quantitative proof ($\eta_p^2 = .376$) of digital platform efficacy in language skill acquisition.

 4. STEM Education & Employability (SDG 4)Relevance: The target population consists of Computer Science undergraduates, addressing the global demand for technically proficient STEM graduates who possess 21st-century workplace communication skills. Connection: If your research examines graduate attributes, soft skills development in technical disciplines, or alignment with United Nations SDG 4 (Quality Education / Target 4.4), this paper presents a model for bridging technical competence and language proficiency.

 5. Quantitative & Quasi-Experimental Methodology in Language TestingRelevance: The research uses a pretest-posttest control group design ($N = 162$), multi-rater scoring with validated rubrics ($ICC = .92$), and ANCOVA for effect size estimation. Connection: If your focus is on research methods, assessment instruments, or statistical modeling in educational research, this study serves as a reference for baseline equivalence testing, inter-rater reliability, and partial eta-squared effect size reporting.

Is the work clearly and accurately presented and does it cite the current literature?

Yes

If applicable, is the statistical analysis and its interpretation appropriate?

Yes

Are all the source data underlying the results available to ensure full reproducibility?

Yes

Is the study design appropriate and is the work technically sound?

Yes

Are the conclusions drawn adequately supported by the results?

Yes

Are sufficient details of methods and analysis provided to allow replication by others?

Yes

Reviewer Expertise:

NA

I confirm that I have read this submission and believe that I have an appropriate level of expertise to confirm that it is of an acceptable scientific standard.

# Reviewer report: Reviewer response for version 1

**DOI:** 10.5256/f1000research.201285.r502023

**Published:** 2026-07-23

**Authors:** Torang Siregar

**Affiliations:** 1 Department of Mathematics Education, PhD Student in Educational Science (Mathematics Education), Universitas Negeri Padang, Padang, West Sumatra, Indonesia; 2 State Institute for Islamic Studies Padangsidimpuan, Padang Sidempuan, Indonesia

This is a methodologically rigorous, well-written, and theoretically significant study that makes meaningful contributions to EOP instruction, culturally responsive pedagogy, and educational technology in higher education. The manuscript is scientifically sound across all evaluation criteria. The authors have demonstrated exceptional transparency through comprehensive data sharing and clear methodological reporting.

**Strengths:**

1. *Strong theoretical framework integrating sociocultural theory and ethnopedagogy*
2. *Rigorous quasi-experimental design with baseline equivalence confirmation*
3. *Validated, multidimensional speaking assessment with excellent psychometric properties*
4. *Large sample size (N = 162) with adequate statistical power*
5. *Transparent data sharing and open access publication*
6. *Clear articulation of limitations and future research directions*
7. *Practical contribution to SDG 4 Quality Education agenda*
8. *Excellent visual presentation of conceptual framework and results*

**Minor Suggestions for Enhancement (Not Required for Acceptance):**

1. **Internationalization:** Briefly define Indonesian-specific terms (e.g., "BAN-PT Level B") for global readers (Page 6)
2. **Replication Enhancement:** Include supplementary materials with sample lesson plans, unit outlines, or specific activity descriptions from the four EOP EMPOWER units to facilitate full replication
3. **Qualitative Supplement:** Acknowledge in limitations that the study's quantitative focus captures learning outcomes but not the processes of engagement; consider mixed-methods follow-up
4. **Ethnopedagogical Specificity:** While the authors note that four of twenty-nine Riau Malay identity values were incorporated, a brief table or description of how each value was operationalized in specific learning activities would strengthen the pedagogical transparency (Page 12)
5. **Technical Detail:** Clarify whether speaking assessments were conducted synchronously (live video) or asynchronously (recorded submissions), as this has implications for speaking anxiety and performance (Page 5)

**Recommendation:** **Accept** - *The manuscript is scientifically sound, well-presented, and makes a valuable contribution to the literature. The minor suggestions above would enhance the manuscript but do not represent critical deficiencies requiring correction.*

**Reviewer's Conclusion:** This study represents a model of rigorous, transparent, and culturally responsive educational research. The integration of EOP with ethnopedagogy through a digital platform is innovative, theoretically grounded, and empirically validated. The work advances both theoretical understanding of sociocultural mediation in language learning and practical pedagogy for STEM education in multilingual contexts. The strong effect sizes and multidimensional speaking improvements provide compelling evidence for the EED Framework's effectiveness and replicability across diverse higher education settings.

Is the work clearly and accurately presented and does it cite the current literature?

Yes

If applicable, is the statistical analysis and its interpretation appropriate?

Yes

Are all the source data underlying the results available to ensure full reproducibility?

Yes

Is the study design appropriate and is the work technically sound?

Yes

Are the conclusions drawn adequately supported by the results?

Yes

Are sufficient details of methods and analysis provided to allow replication by others?

Yes

Reviewer Expertise:

Mathematics Education; STEM Education; RME; AI in Education, Mathematics Teaching and Learning; Educational Technology; GeoGebra-Assisted Learning; Artificial Intelligence in Education; Mathematical Literacy; Problem Solving; Critical Thinking; Numeracy; Curriculum and Instruction; Teacher Education; Research & Development; Higher Education; Social Science.

I confirm that I have read this submission and believe that I have an appropriate level of expertise to confirm that it is of an acceptable scientific standard.
